# Modeling the Overalternating Bias with an Asymmetric Entropy Measure

**DOI:** 10.3389/fpsyg.2016.01027

**Published:** 2016-07-06

**Authors:** Giorgio Gronchi, Marco Raglianti, Stefano Noventa, Alessandro Lazzeri, Andrea Guazzini

**Affiliations:** ^1^Department of Neuroscience, Psychology, Drug Research and Child's Health - Section of Psychology, University of FlorenceFlorence, Italy; ^2^Formerly affiliated with the BioRobotics Institute, Scuola Superiore Sant'AnnaPisa, Italy; ^3^Center for Assessment, University of VeronaVerona, Italy; ^4^Department of Information Engineering, University of PisaPisa, Italy; ^5^Department of Sciences of Education and Psychology, University of FlorenceFlorence, Italy

**Keywords:** randomness perception, overalternating bias, asymmetric entropy, Renyi's entropy, Marcellin's entropy, Shannon's entropy, Differential Evolution algorithm

## Abstract

Psychological research has found that human perception of randomness is biased. In particular, people consistently show the overalternating bias: they rate binary sequences of symbols (such as Heads and Tails in coin flipping) with an excess of alternation as more random than prescribed by the normative criteria of Shannon's entropy. Within data mining for medical applications, Marcellin proposed an asymmetric measure of entropy that can be ideal to account for such bias and to quantify subjective randomness. We fitted Marcellin's entropy and Renyi's entropy (a generalized form of uncertainty measure comprising many different kinds of entropies) to experimental data found in the literature with the Differential Evolution algorithm. We observed a better fit for Marcellin's entropy compared to Renyi's entropy. The fitted asymmetric entropy measure also showed good predictive properties when applied to different datasets of randomness-related tasks. We concluded that Marcellin's entropy can be a parsimonious and effective measure of subjective randomness that can be useful in psychological research about randomness perception.

## 1. Introduction

### 1.1. Inductive reasoning and subjective randomness

Explaining how people make inductive reasoning (e.g., inferring general laws or principles from the observation of particular instances) is a central topic within the psychology of reasoning. In particular, perception of randomness is a key aspect of these inferential processes. Perceiving a situation as non-random requires some kind of subjective explanation which entails the onset of inductive reasoning (Lopes, 1982). On the contrary, if the phenomenon is seen as a mere coincidence, the observer does not hypothesize any explanation. For example, during World War II the German air force dropped on London V1 bombs: many Londoners saw particular patterns related to the impacts and consequently they developed specific theories about German strategy (e.g., thinking that poor districts of London were privileged targets). However, a statistical analysis of the bombing patterns made after the end of the war revealed that the distribution of the impacts was not statistically different from an actual random pattern (Hastie and Dawes, 2010). The opposite mistake happens when an observer fails to detect a regularity, thus attributing to chance a potential relation noticed (Griffiths, 2004): before Halley, no one had ever thought that the comets observed in 1531, 1607, and 1682 were the very same comet (Halley, 1752). Since 1950, many psychological studies have been devoted to investigate randomness perception and production: an important result is that people's intuitive understanding of randomness in binary sequences is biased toward an over-alternation between different possible outcomes (the so-called overalternating bias).

Given the importance of having a viable and flexible measure of subjective randomness, this study aims to evaluate how different kinds of entropy measures can predict judgments about sequence randomness. In particular, within the context of data mining and growing decision trees, an asymmetric measure of entropy has been developed (Marcellin et al., 2006). Such measure has proven to be very useful in dealing with unbalanced classes in medical and economic decisions. Nonetheless, such asymmetric entropy measure might also be beneficial in cognitive domains. In this paper we investigate its usefulness in order to model the overalternating bias.

### 1.2. The overalternating bias

From a formal point of view, randomness is still an elusive concept and a shared definition has yet to be established. A variety of efforts have been sustained in order to provide a formal measure of randomness within mathematics, physics, and computer science (Li and Vitányi, 1997; Volchan, 2002). Despite the lack of a clear and shared normative criterion, psychologists have been investigating extensively people's subjective sense of randomness. Usually participants' responses are compared to sampling distributions of statistics that characterize the stimuli. This strand of research has employed classically two types of tasks: production tasks and perception tasks. In the former, participants are asked to generate the outcomes of a random mechanism, for example simulating the results of tossing a fair coin. On the contrary, in perception tasks participants have to rate how much random on a Likert scale the stimulus is (commonly a string of binary elements) or to categorize the stimulus on the basis of the generating source (e.g., has the sequence been produced by a random or a non-random mechanism?).

Despite some methodological issues that characterize the psychological investigation of randomness (Nickerson, 2002), the basic finding of generation and perception of random binary strings (and two-dimensional grids of binary elements) is the overalternating bias: people identify randomness with an excess of alternation between symbol types compared to the normative criterion employed. In other terms, those sequences which actually present the modal number of alternations expected by chance are not perceived as maximally random because they contain too long runs of the same element. Falk and Konold (1997) made a series of randomness perception experiments that clearly showed such an overalternating bias. They employed 21-elements strings composed by two symbols, Xs and Os, such as XXXX…OOOO. The alternation rate of such sequences can be defined through the probability of alternation [*P*(*A*)] statistics: this value is defined as the ratio between the number of actual transitions and the number of total transitions in the sequence. More formally, for strings of length *n* and with a number of runs (i.e., unbroken subsequences) *r*, the probability of alternation is

(1) $$\begin{array}{l} P\left(A\right)=\frac{r-1}{n-1} \end{array}$$

Falk and Konold (1997) employed as a normative criterion to quantify the randomness of a sequence the second order entropy of the sequence computed with the classical Shannon entropy (Shannon, 1948). Such measure is based on the relative frequencies of the ordered pairs of symbols, called digrams (in the example, XO, OX, XX, and OO); in particular, it quantifies the new information (in bits) contributed by the second member of the pair. It is possible to define second order entropy as the difference between the entropy of the digrams and the first order entropy (Attneave, 1959):

(2) $$\begin{array}{l} H_{2}=H_{\left(digram\right)}-H_{1} \end{array}$$

First order entropy can be computed through the classical Shannon formula:

(3) $$\begin{array}{l} H_{1}=\sum p_{i}log\frac{1}{p_{i}} \end{array}$$

Where *p*_*i*_ is the probability of the symbol *i*. Similarly, the entropy of the digrams can be obtained on the basis of the probability of the ordered pairs of symbols:

(4) $$\begin{array}{l} H_{\left(digram\right)}=\sum p_{\left(digram\right)}log\frac{1}{p_{\left(digram\right)}} \end{array}$$

**Figure 1:**
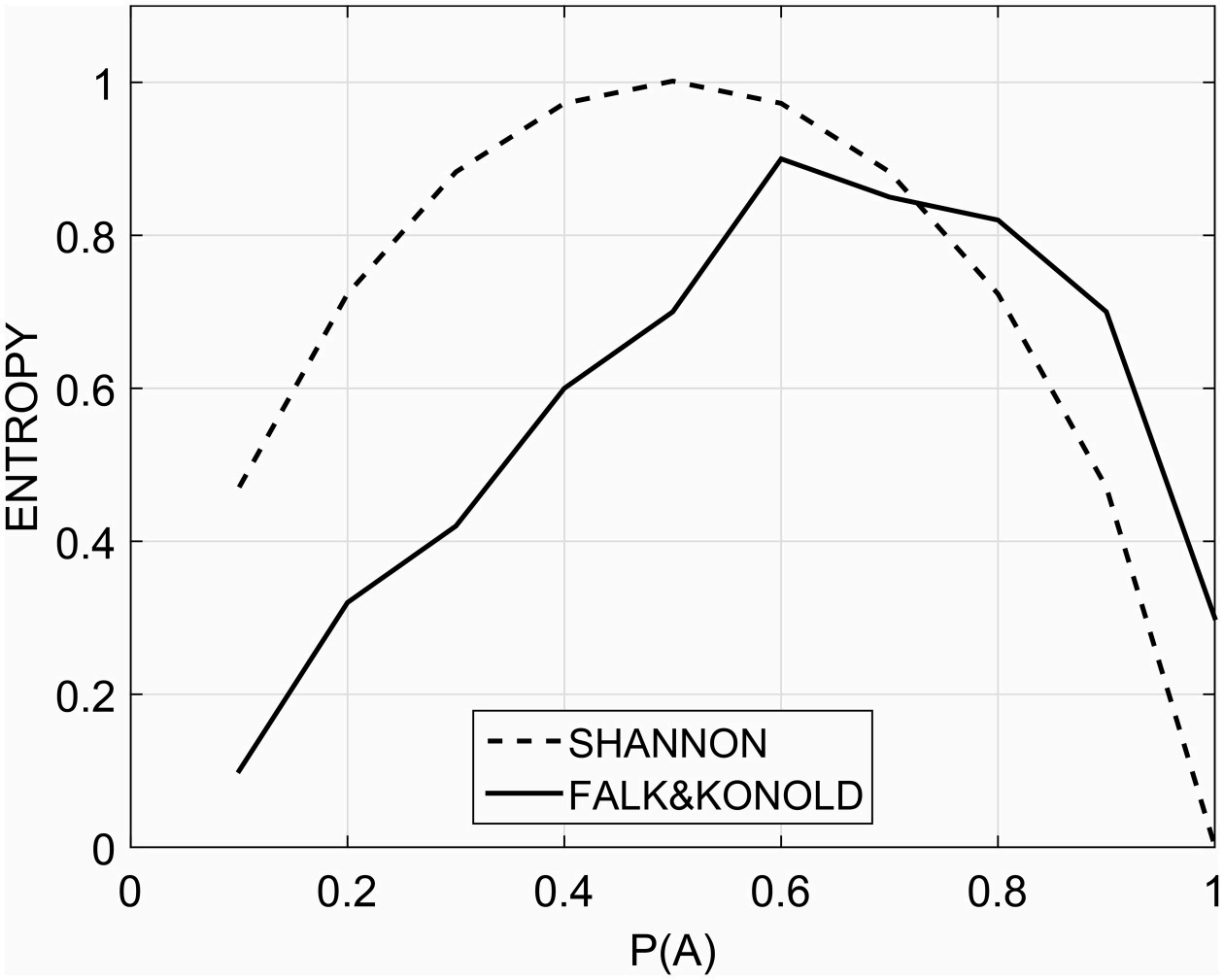
**The empirical subjective randomness measured by Falk and Konold (solid line) and the second order entropy computed by Shannon's formula (dashed line)**.

The relationship between the probability of alternation and the second order entropy is a symmetrical, unimodal reversed-U curve with a maximum in correspondence of a probability of alternation value of 0.5 (Figure 1). However, while measuring the subjective randomness rating of binary strings by manipulating the probability of alternation, participants indicated that the most random rated sequences were the ones with a probability of alternation of about 0.7. The resulting function is an asymmetrical U-reversed relationship negatively skewed (Figure 1). This is a clear example of overalternating bias. The empirical function of subjective randomness is different from the function that is obtained by computing the second order entropy as a normative criterion of randomness.

For example, strings such as XXXOXXOOXOO [*P*(*A*) = 0.5] are rated less random than OXXOXXOXOOX [*P*(*A*) = 0.7] although this is not true from a normative point of view. This kind of result is very robust and it has been found in a variety of studies. Reviewing the literature, Falk and Konold (1997) found that the sequences rated as most random ranged from a *P*(*A*) = 0.57 to 0.8. Nevertheless, these works employed a variety of stimuli (strings or two-dimensional grids), different sizes of the set of stimuli and a variety of task instructions (such as select the most random sequence or rate their randomness on a Likert scale).

### 1.3. Measuring subjective randomness

Within psychology literature, two main measures of subjective randomness for strings of symbols have been proposed: Difficulty Predictor (DP) (Falk and Konold, 1997) and the model of Griffiths and Tenenbaum (2003, 2004). Both measures try to quantify the complexity of a sequence in order to compute a score of subjective randomness in accordance with empirical data on human judgments.

The DP score is computed by counting the number of runs (any uninterrupted subsequence of the same symbol), and adding twice the number of subsequences in which the symbols alternate. For example, the sequence XXXOOOXOXO is composed by a run of Xs, a run of Os, and an alternating sub-sequence (XOXO), for a total value of DP equal to 4. If there are multiple ways to segment the string, DP is calculated on the partition that results in the lowest score. DP correlates very highly with randomness judgments and a variety of related tasks. However, DP is a parameter-free score and it is not possible to use it to quantify how subjective randomness changes in different conditions (e.g., by fitting DP to data obtained with different tasks to investigate the variation of the parameters). Moreover, as Griffiths and Tenenbaum (2003) observed, DP is not able to account for subjective randomness with strings of different length: for example, XXXXXXXXXXXOOXO and XXXOOXO have the same value of DP (4) but clearly the long uninterrupted run of Xs of the former provides a stronger evidence for some kind of regularity.

Griffiths and Tenenbaum instead employed the Bayesian framework to develop a probabilistic model of the overalternating bias (Griffiths and Tenenbaum, 2003, 2004). The randomness perception task is addressed in terms of the statistical problem of Bayesian model selection: given a string, it has to be inferred whether the process that generated it was random or regular. From a rational point of view, the probability of obtaining a specific binary string given a random generating process is constant and equal to ${\left(\frac{1}{2}\right)}^{k}$ where *k* is the number of elements of the string. Conversely, the probability of obtaining that particular sequence given a regular generating process is computed by means of a Hidden Markov Model (HMM): through the parameters of the model it is possible to determine regularities that people perceive when judging the randomness of a binary sequence. In sum, the authors showed how through a Bayesian framework is possible to model the perceived randomness of binary sequences and its sensitivity to motif repetition and other kind of regularities (such as various types of symmetry). By means of these models it is possible to predict accurately human judgments, including the overalternating bias. Depending on the kind of regularities that can be detected, it is possible to specify models of increasing complexity (from 4 to 8 parameters). Overall, results show that the model with the highest number of parameters account better for observed data and that such parameters vary coherently with different experimental conditions (Griffiths and Tenenbaum, 2003, 2004). This model has a very high number of parameters and it is deeply grounded in a specific psychological theoretical framework (the Bayesian probabilistic perspective), greatly complicating its use for those who do not adhere to such perspective.

DP and the Griffiths and Tenenbaum model are highly correlated and they are both able to account very well for randomness judgments. The aim of the present work is then to explore the possibility of modeling randomness judgments with a parsimonious, parameter-based model not grounded into any specific psychological framework. To this purpose, we focused on some of the various measures of entropy proposed within mathematics, physics, and information sciences.

## 2. Measures of uncertainty: Renyi's entropy and the asymmetric entropy of Marcellin

As we have seen in Section 1.2, information theory has provided a normative criterion (the second order entropy) to quantify the uncertainty of strings of characters which are employed in experimental psychology.

Moving however from Shannon's definition and relaxing some of its assumptions, others generalized versions and families of information entropies have been obtained by authors like Rényi (1961), Beck and Cohen (2003), Tsekouras and Tsallis (2005), and Marcellin et al. (2006). Given indeed a distribution over a set of events P = (*p*_1_, …, *p*_*N*_), information entropy *H* was derived as a measure of the choice involved in the selection of an event (or in the uncertainty of the outcome), by requiring continuity in the events *p*_*i*_, monotonicity in *N* when equiprobability holds, and that if a choice can be broken down into two successive choices, the original *H* should be the weighted sum of the individual values of *H* (Shannon, 1948). By relaxing, for instance, the third requirement to a less restrictive form of additivity, in which not only weighted sums are allowed but more general additive functions, Rényi (1961) obtained the following generalization

(5) $$\begin{array}{l} H_{\alpha }\left(P\right)=\frac{1}{1-\alpha }\log\left(\overset{N}{\underset{i=1}{\sum }}p_{i}^{\alpha }\right) \end{array}$$

where α is a non-negative integer and the scaling factor $\frac{1}{1\;-\;\alpha }$ is given so that for a uniform distribution U it always holds *H*_α_(U) = log*N* for all values of α. The previous expression is defined as the Renyi entropy of order α of a distribution P and it is widely used in statistics, biology, and in quantum information theory as a measure of entanglement. It is a bounded, continuous, non-increasing and non-negative function of α, it is concave for α ≤ 1 and it loses concavity over the critical value α_*c*_ which is a function of *N*. On passing, notice also that Renyi's entropy can be given an interpretation in terms of p-norm on a simplex in *N* dimensions. Most of all, it obeys additivity meaning that given two distributions P and Q, it holds:

$$\begin{array}{l} H_{\alpha }\left(P*Q\right)=H_{\alpha }\left(P\right)+H_{\alpha }\left(Q\right) \end{array}$$

Interestingly, entropy (5) encompasses several measures of uncertainty such as Hartley's entropy, quadratic entropy, min entropy, and the Shannon entropy. Indeed, changes in the parameter α imply that probabilities are sort of weighted. More in detail, for α = 0 it returns the Hartley (max) entropy *H*_0_(P) = log*N*, so that lower values of α move toward equiprobability; if instead α = 2 it returns the quadratic (collision) entropy $H_{2}(P)=-\log(\sum_{i=1}^{N}p_{i}^{2})$; while in the limit α → ∞ it returns the min entropy $H_{\infty }\left(P\right)=\underset{i}{\min}\left(-\log p_{i}\right)$ so that higher values of α shift the attention toward the event with maximum probability. Finally, in the limit α → 1, by means of L'Hopital's rule, one can show that Renyi's entropy becomes exactly Shannon's entropy, which is the only limit in which the chain rule (or glomming formula) for conditional probability is satisfied.

Alternatively, one might characterize a generic measure of entropy (including Renyi's) as a non-negative, symmetric and strictly concave function, which is also bounded between a minimum (usually zero, attained when there is one *p*_*k*_ = 1 while all others *p*_*i*_ = 0 for *i* ≠ *k*) and a maximum (attained for the uniform distribution). Within several fields like medicine, marketing, and fraud detection, however, two assumptions from the previous set can become critical: namely, the symmetrical behavior with respect to different permutations of the probabilities, and the association of the maximum entropy with the uniform distribution (which is essentially the Laplace's principle of indifference). Entropy measures are indeed often employed in learning tasks and, in particular, in growing decision trees, in order to assign a leaf of the tree to a specific class by means of suitable splitting rules. Marcellin et al. (2006) noticed that in these cases a symmetric measure of uncertainty can be deceiving since not necessarily the different classes are balanced, meaning that their distribution is not a priori uniform. Moreover, the meaning of detecting a particular class can vary: for example, predicting a wrong disease (a false positive) has different consequences than missing a disease (a false negative), which reflects in non-equal misclassifications costs. In order to overcome these limits, an asymmetrical measure of entropy was proposed:

(6) $$\begin{array}{l} H_{W}\left(P\right)=\overset{N}{\underset{i=1}{\sum }}\frac{p_{i}\left(1-p_{i}\right)}{\left(1-2w_{i}\right)p_{i}+w_{i}^{2}} \end{array}$$

where W = (*w*_1_, …, *w*_*N*_) is the worst distribution for which the maximum value is attained. Such a measure of entropy is non-negative, asymmetric (symmetry is restored if W is uniform) and it is bounded between zero and a maximum.

## 3. Fitting Renyi's and Marcellin's entropies to randomness judgements

### 3.1. Materials and methods

#### 3.1.1. Rationale of the study

Given the properties of Marcellin's asymmetric entropy, such measure might represent a suitable tool to model the overalternating bias. The most notable feature of Marcellin's entropy is that the most uncertain distribution must be estimated from data if not a priori available. This feature should reflect that an asymmetry in the distribution entropy for the probability of alternation would imply that the maximum randomness is actually perceived when the frequencies of alternating and non-alternating digrams are not equal. In our specific case it is expected that maximum randomness is perceived when alternating digrams exceed non-alternating ones. On these basis, we fitted the second order entropy with Marcellin's employing four parameters: *w*_*OO*_, *w*_*XX*_, *w*_*XO*_, *w*_*OX*_. The first couple is related to uniform digrams, whereas the second couple of parameters is related to alternating digrams. Given that XX and OO should be equivalent from a psychological point of view (as well as XO and OX), we constrained the corresponding parameters to be close to each other (see Section 3.2.1, Equations (7) and (8) for further details).

Fitting Marcellin's entropy to data, we expect that *w*_*XO*_ and *w*_*OX*_ will be comprised between 0.5 and 1, thus maximally contributing to the overall entropy of the sequence when the alternating digrams are more likely to appear. Their contribution to the sequence's entropy should be reduced approaching a probability of 1.0 for alternating digrams, since it represents a completely alternating sequence, such as XOXOXOXO, that doesn't result in a high subjective randomness. On the contrary, we expect that *w*_*OO*_ and *w*_*XX*_ will be comprised between 0 and 0.5 suggesting a high subjective randomness when an observer sees a low proportion of uniform digrams. Similarly to the previous case, the parameters *w*_*OO*_ and *w*_*XX*_ must be higher than 0 because a complete absence of uniform digrams does not suggest an high subjective randomness (as in the XOXOXOXO string).

We compared the fit of Marcellin's measure of entropy with the second order entropy computed with Shannon formula (as a reference) and with Renyi's entropy (because it encompasses several measures of uncertainty). We fitted these three measures of entropy to the 10 mean points of subjective ratings observed in Falk and Konold's experiment (Falk and Konold, 1997). Finally, by employing the parameters of Marcellin's entropy estimated with these means, we compared the correlations between such measures and other datasets of random judgments (obtained by Gronchi and Sloman, 2009), as well as DP and Griffiths and Tenenbaum model predictions.

#### 3.1.2. Target values

We used Falk and Konold's (1997) results to fit the models. As described before, in that work the authors asked to “rate each sequence on a scale of 0 to 10 according to your intuition of how likely it is that such a sequence was obtained by flipping a fair coin.” They employed forty strings comprised in four alternative sets of 21 binary symbols (O and X). Each set was composed by 10 sequences with a probability of alternation ranging from 0.1 to 1 (in intervals of 0.1). Half of the sequences had 11 Xs and 10 Os and other half 10 Xs and 11 Os. For each value of probability of alternation, the mean randomness rating was computed obtaining a set of 10 points. We employed those values as a target function for the fitting problem.

#### 3.1.3. Parameter fitting

In this Section we present the approach followed to find the optimal parameters of Renyi's and Marcellin's entropy models to fit the target function. First, we adopted the Euclidean distance between the target function and the *H*_2_ of model (2) as a fitness measure to quantify the goodness of the solution. More precisely, we wanted to find (i) an optimal alpha for Renyi's entropy and (ii) an optimal set of weights for Marcellin's entropy. To adapt the parameters for the minimization of a fitness measure is a classic optimization problem.

In several domains of application researchers employ *search* methods, i.e., algorithms that test solutions of the problem until a satisfactory condition is met. These methods are usually adopted because they are “black box” approaches, thus they are not based on the formal properties of the quality function. As a consequence, convergence to the optimal solution is not guaranteed, thus we need statistical measures to identify the goodness of the solution.

We used the Differential Evolution (DE) algorithm (Storn and Price, 1997) to solve our problem. DE has been recently used by researchers for several optimization problems because of its performance in unimodal, multimodal, separable, and non-separable problems (Das and Suganthan, 2011). DE is a population based algorithm, in which a member of the population is a vector that represents the parameters of the model. The size *N* of the population is usually between 2 and 20 times the number of elements of the vector. A large *N* increases the time to compute a new generation, but speeds up the convergence of the algorithm. To balance the two aspects we use *N* = 20. Each member of the population is evaluated via the fitness measure previously described. DE iteratively improves the population selecting a target member *v*_*ta*_ and making a comparison with a trial member *v*_*tr*_. The trial is generated in two steps: the mutation and the crossover. In the mutation, three random vectors *v*_1_, *v*_2_, *v*_3_, from the population, excluded the target, are combined in a mutant vector: *v*_*m*_ = *v*_1_ + *F* · (*v*_2_ − *v*_3_), where *F* ∈ [0, 2] is the *differential weight*. In the crossover, given the *crossover rate CR* ∈ [0, 1], the trial member is computed randomly selecting an element either from the target or the mutant with probability 1−*CR* and *CR* respectively. Finally, DE compares the fitness measure of the target and the trial vectors. The one with the best value remains in the next generation and the other is discarded. The interested reader can refer to Cimino et al. (2015) for further information on the parameterization and the behavior of DE.

To identify the proper values of *F* and *CR* we ran the algorithm 10 times for 100 generations with the following combinations of parameters: *F* from 0.1 to 2 in steps of 0.1, and *CR* from 0.1 to 0.9 in steps of 0.1. We considered two criteria: (i) the algorithm converges to the best fitness and (ii) the least average number of generations needed to find the solution. The convergence condition is met when at least one of the member fitness is lesser than the best fitness among all trials increased by 1%.

### 3.2. Results

#### 3.2.1. Parameter fitting's results

The best set of *CR* and *F* is 0.9 and 0.6, respectively. With this setting we ran DE for 100 times and in Table 1 we summarized the results. For Renyi's model optimization, all the runs converged toward the same solution. For Marcellin's model 95% converged toward the best solution found among all trials. Only 5% converged toward a local minimum. However, the worst solution found by DE with Marcellin's model is still better than the best found with Renyi's model.

**Table 1:** **Best parameter tuning for different entropy models and their respective best and worst fitness measures (and percentage of convergence) found by DE**.

**Entropy**	**Best parameters tuning**	**Fitness measure value (% convergence)**	
		**Best**	**Worst**
Shannon	–	0.949	x
Renyi	α = 2.37	0.875 (100%)	(0%)
Marcellin	*w_OO_* = 0.33, *w_XO_* = 0.68, *w_OX_* = 0.69, *w_XX_* = 0.30	0.728 (95%)	0.755 (5%)

As described in Section 3.1.1, Marcellin's model is subjected to two constraints: the first binds the weights *w*_*OO*_ and *w*_*XX*_ to be close to one another, and the second binds *w*_*XO*_ to *w*_*OX*_. We implemented the constraint binding the relative distance between the weights *w*_*OO*_ and *w*_*XX*_ to be lesser than or equal to 0.1 as in Equation (7) [same for weights *w*_*OX*_ and *w*_*XO*_ in Equation (8)]. This implementation restricts the search space of DE, i.e., the trial vectors violating at least one of the constraints are discarded and a new one is instead generated. These constraints reflect that the uniform digram XX should be equivalent to OO from a psychological point of view (as well as XO should be equivalent to OX).

(7) $$\begin{array}{r} 2\cdot \frac{\left|w_{OO}-w_{XX}\right|}{w_{OO}+w_{XX}}\leq 0.1 \end{array}$$

(8) $$\begin{array}{r} 2\cdot \frac{\left|w_{OX}-w_{XO}\right|}{w_{OX}+w_{XO}}\leq 0.1 \end{array}$$

**Figure 2:**
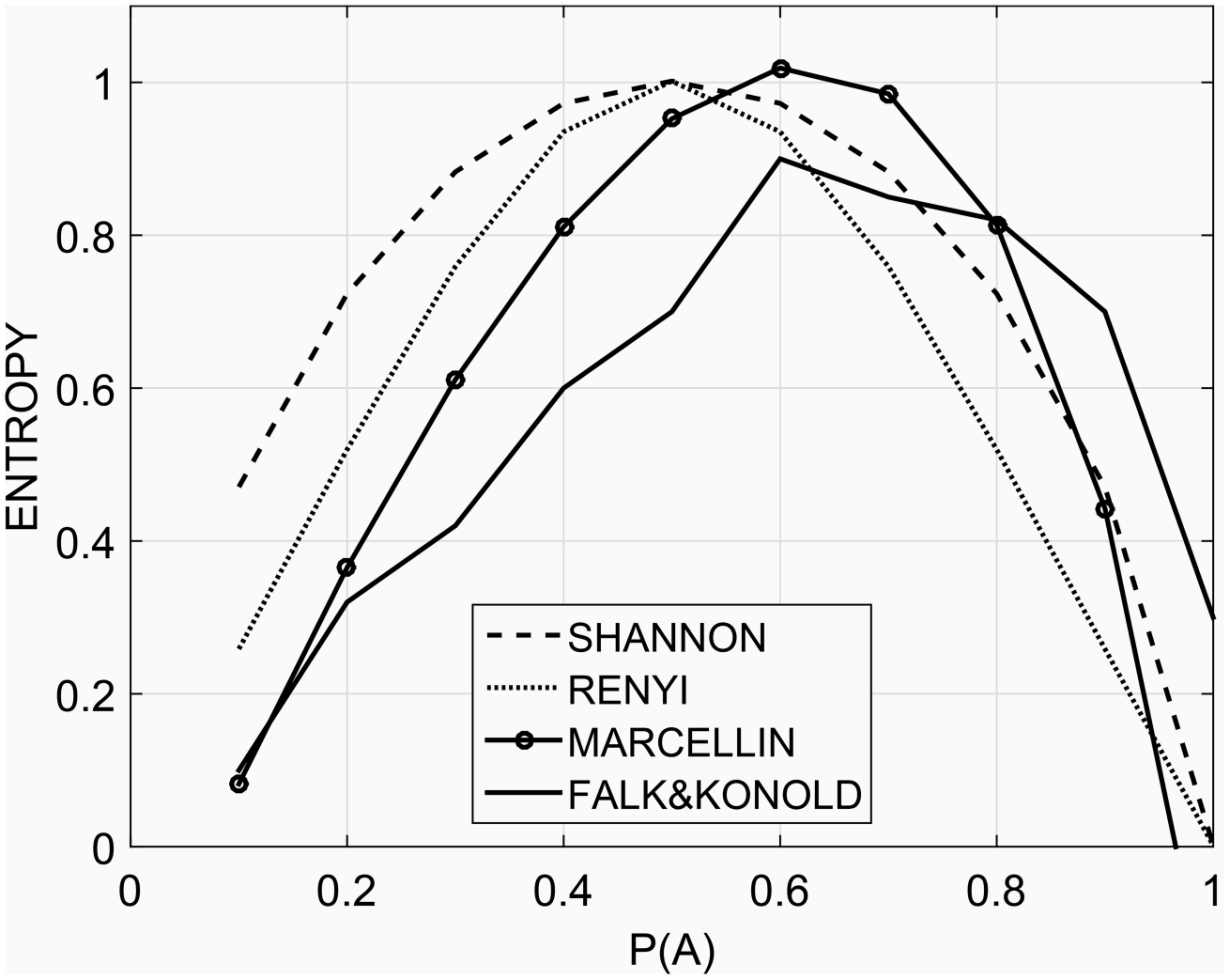
**The target function (solid) is the empirical data from Falk and Konold**. The entropies are respectively computed by Shannon (dashed), Renyi (dotted), and Marcellin (solid with circles) formulas after parameter fitting.

In line with our expectations, we found that *w*_*OO*_, *w*_*XX*_ were comprised between 0 and 0.5 whereas *w*_*XO*_ and *w*_*OX*_ were comprised between 0.5 and 1 (Table 1). Figure 2 shows the best fit of the empirical data from Falk and Konold (target function, solid line) by the three entropy models. While Shannon's (dashed line) and Renyi's (dotted line) models show a symmetrical curve centered in *P*(*A*) = 0.5, Marcellin's model (solid with circles) shows an asymmetrically right-skewed shape more closely approximating Falk and Konold's data.

#### 3.2.2. Validation of Marcellin's entropy as a measure of subjective randomness

Given the parameters obtained for Marcellin's entropy in the previous section, we computed its Pearson product-moment correlations with both the results of other randomness task experiments and other two subjective randomness scores (DP and Griffiths and Tenenbaum model). Griffiths and Tenenbaum parameters were estimated in a separate experiment (Gronchi and Sloman, 2009). The two datasets that we employed for validation were based on a categorization task: participants observed sequences of eight binary elements (Heads and Tails). All the possible sequences of eight elements are 256, but since there are two sequences for each different configuration of elements (e.g., TTTTTTTT is equivalent to HHHHHHHH), only half of them were employed (128). Participants were instructed that they were going to see sequences which had either been produced by a random process (flipping a fair coin) or by some other process in which the sequences of outcomes were not random, and they had to classify these sequences according to what they believed to be their source (random or regular). For each sequence, the authors computed the proportion of participants that classified the strings as random (thus, obtaining a value between 0 and 1). Experiment A (Gronchi and Sloman, 2009) was conducted without measuring reaction times of participants whereas in experiment B (Gronchi and Sloman, 2009) participants were required to respond as fast as they could and reaction times were recorded.

**Figure 3:**
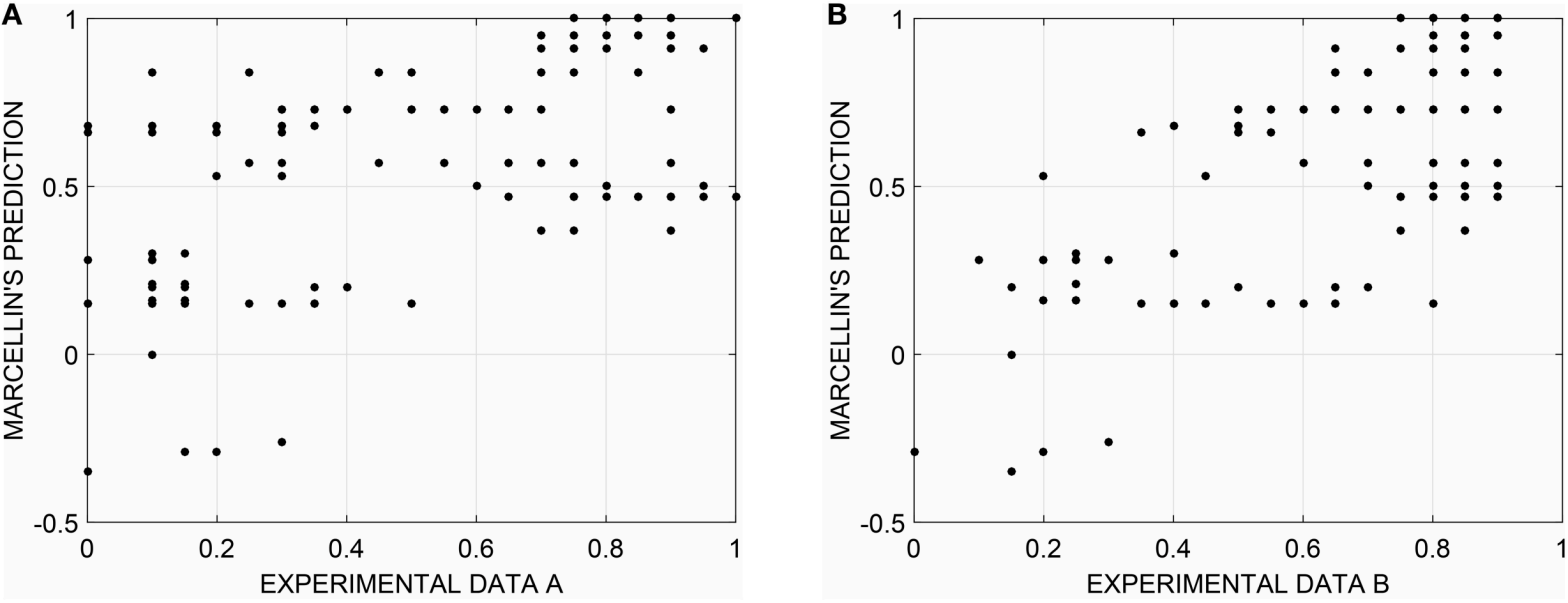
**Relationship between the percentage of random responses for the set of 128 sequences of Experiment A (A) and B (B) and their Marcellin's entropy (with fitted parameters, Table 1)**. Pearson's *r* = 0.60 and *r* = 0.67, respectively.

The relationship between the percentage of random responses given to each of the 128 sequences of Experiment A (Figure 3A) and B (Figure 3B) and their Marcellin's entropy (with fitted parameters) resulted in a Pearson's *r* = 0.60 and *r* = 0.67 respectively (Figure 3). Correlations were also computed between the percentage of random responses and the other corresponding measures of subjective entropy (DP and Griffiths and Tenenbaum, Table 2). Experiment A results were highly correlated to all measures of subjective randomness: it was observed a correlation value equal to 0.60 for Marcellin, 0.67 for DP, and 0.76 for Griffiths and Tenenbaum model. With regard to experiment B, correlation values were 0.67, 0.73, and 0.80 for Marcellin, DP, and Griffiths and Tenenbaum, respectively.

**Table 2:** **Pearson product-moment correlations of Experiment A and B results with different subjective randomness scores (Marcellin's Entropy, Difficulty Predictor, Griffiths and Tenenbaum's model)**.

	**Marcellin**	**DP**	**G&T**
Experiment A	0.60	0.67	0.76
Experiment B	0.67	0.73	0.80

## 4. Discussion and conclusions

In this paper we investigated the potentiality of Marcellin's asymmetric entropy for predicting randomness judgments and the overalternating bias. Fitting Marcellin's entropy to randomness rating, we observed a better fit compared to subjective randomness measures based on classical Shannon's entropy and on Renyi's entropy, which represents a generalized form of such measures of uncertainty comprising many different kinds of symmetric entropies. The estimated parameters for Marcellin's are coherent with the overalternating bias: the overabundance of alternating substrings corresponds to a high value of subjective randomness (compared to the Shannon entropy criterion which provides an equal proportion of these substrings). In the same way, the lack of uniform substrings indicates a high value of subjective randomness. Frequencies are about 68% for the alternating substrings and 30–32% for the uniform ones. Differently from Marcellin's, the other entropy measures are symmetric around the equipartition of the events, so they are unable to account for the overalternating bias.

We validated the asymmetric-entropy-based measure of subjective randomness correlating it with different datasets and other subjective randomness scores (DP and Griffiths and Tenenbaum model). As expected by previous literature (Falk and Konold, 1997; Griffiths and Tenenbaum, 2003, 2004), DP and Griffiths and Tenenbaum's model were highly correlated with empirical judgments. Although such correlations were higher compared to Marcellin's entropy, the Pearsons's *r*-values of the latter are indeed high, with a minimum value of 0.60. Given the very noisy nature of these experiments, this result confirms the potentiality of Marcellin's asymmetric entropy for modeling randomness judgments.

Marcellin's entropy may thus represent a viable alternative to DP and Griffiths and Tenenbaum's measure. As a matter of fact, there can be some cases in which such measures are of limited use. Being a parameter-free measure, the simplicity and the lack of any theoretical framework of DP are together its strengths and weaknesses. On the one hand, DP can easily be computed for quantifying subjective randomness without any fitting procedure. On the other hand, DP cannot be used to investigate how different factors can affect randomness judgments and the overalternating bias. Moreover, DP is a coherent measure only when computed over strings of the same length because it is not affected by the length of uniform subsequences of outcomes (such as XXX or XXXXX) in a sequence. Indeed, the subjective randomness of a uniform subsequence decreases as the length of the substring increases.

On the contrary, the measure of Griffiths and Tenenbaum (2003, 2004) has several parameters and it is theoretically grounded in the Bayesian probabilistic framework. Its complexity is counterbalanced by the possibility to model what are the kinds of regularities (motifs repetition and length, symmetries, duplications) that influence randomness judgments. So, by using the Griffiths and Tenenbaum (2003, 2004) model it is possible to investigate how different factors can alter subjective randomness and hypothesis about randomness perception. For example, employing this model, Hsu et al. (2010) explored the hypothesis that the regularities detected in two-dimensional binary visual arrays (and the resulting randomness evaluation) is affected by the statistical structure of our visual world. Furthermore, the model of Griffiths and Tenenbaum was conceived combining the rational statistical inference approach with the algorithmic information theory^1^. Being grounded into well-known mathematical and information science theories, their measure can exploit the advantages of being expressed in formal terms. Significantly, the authors demonstrated how the Bayesian probabilistic modeling approach (that has been proven to account for many psychological phenomena) is also able to address the domain of randomness perception. However, this aspect can also be a limiting factor because the use of the Bayesian approach in psychology is still a controversial issue (Bowers and Davis, 2012) and there is no unanimously accepted opinion about its application in modeling cognitive processes.

Marcellin's entropy as a measure of subjective randomness stands in a middle ground between DP and Griffiths and Tenenbaum's model. Differently from DP, Marcellin's entropy is a parameter-based measure but it is more simple and parsimonious than Griffiths and Tenenbaum's model. Marcellin's entropy can be employed to quantify how much randomness judgments are distorted toward the overalternating bias and thus it is possible to investigate how different factors may affect participants' responses. However, the greater parsimony of Marcellin's measure entails the impossibility to assess the kinds of regularities that influence the judgments. In sum, Marcellin's entropy is a measure, defined in formal terms and drawn from current data mining literature, whose parameters appear to suitably characterize the bias in our perception and does not require to accept the Bayesian approach as a theoretical reference framework. Such measure can be considered a suitable alternative to DP and Griffiths and Tenenbaum's model to quantify subjective randomness in future psychological studies.

## Author contributions

GG conceived of the study, participated to simulation, drafted and discussed with MR and SN the initial manuscript, and corrected and organized the subsequent versions. MR assisted in drafting the initial manuscript and developed the MATLAB scripts for carrying out the initial analysis. SN wrote Section 2 and contributed to the formal analysis of the comparisons among measures. AL implemented the Differential Evolution algorithm carrying out the fitting procedures. AG participated to simulation and data analyses and reviewed the work. All authors approved the final manuscript as submitted.

## Funding

This work was supported by EU Commission (FP7-ICT-2013-10) Proposal No. 611299 SciCafe 2.0.

### Conflict of interest statement

The authors declare that the research was conducted in the absence of any commercial or financial relationships that could be construed as a potential conflict of interest.

## Footnotes

^1^Within algorithmic information theory, the concept of Kolmogorov's complexity has been used to mathematically define the randomness of sequences of outcomes (Li and Vitányi, 1997).
